# Association of late-life depression with cognitive impairment: evidence from a cross-sectional study among older adults in India

**DOI:** 10.1186/s12877-021-02314-7

**Published:** 2021-06-15

**Authors:** T. Muhammad, Trupti Meher

**Affiliations:** International Institute for Population Sciences, 400088 Mumbai, Maharashtra India

**Keywords:** Depression, Cognitive impairment, Older adults, India

## Abstract

**Background:**

Late-life depression (LLD) is considered as a prodrome to dementia and plays a major role in the development of long-term cognitive disabilities. We aimed to estimate the prevalence and correlates of LLD and cognitive impairment and to explore their associations among older adults in India.

**Methods:**

Data for this study was derived from the Longitudinal Ageing Study in India (LASI) Wave 1 (2017-18). The total sample included 31,464 (15,098 male and 16,366 female) older individuals aged 60 years and above. Cognitive impairment measured from various domains derived from the cognitive module of the Health and Retirement Study (HRS), and major depression measured by the CIDI-SF (Composite International Diagnostic Interview- Short Form) were the outcome variables. Descriptive, bivariate, and multivariable analyses were performed to fulfill the objectives of the study.

**Results:**

The overall prevalence of LLD and cognitive impairment for the current sample was 8.7% and 13.7 % respectively. Among older individuals who have rated their health status as poor were 2.59 times more likely to suffer from LLD [OR: 2.59, CI: 2.24–2.99] as compared to their counterparts. The older adults who had difficulty in activities of daily living (ADL) and instrumental activities of daily living (IADL) were 74% and 69 % more likely to suffer from LLD. Similarly, older adults who were depressed had higher odds of cognitive impairment [OR: 1.22, CI: 1.01–1.48] compared to their counterparts. Also, older adults who were depressed and belonged to rural areas were 2.58 times [AOR: 2.58, CI: 1.95–3.41] more likely to be cognitively impaired than those who were not depressed and resided in urban areas.

**Conclusions:**

Depression is linked to an increased risk of cognitive decline and dementia; therefore, failing to diagnose and treat LLD in later life may have significant health implications. Moreover, treatment under the care of a cognitive neurologist or geriatric psychiatrist is recommended for people with LLD and cognitive disability due to both the disorders' complex existence.

**Supplementary Information:**

The online version contains supplementary material available at 10.1186/s12877-021-02314-7.

## Introduction

In an aging population, cognitive impairment is not only a major risk factor for ill health, but it also places a significant burden on public health [1]. It is common in late life and may be caused by aging and associated physical or psychological disorders [2]. Cognitive impairment and dementia result in a gradual and devastating reduction in most physical abilities, functional independence, and social relationships. The global dementing population has been growing and is expected to reach 131.5 million by 2050 [3]. Further, dementia is expected to rise from 58 % in 2010 to 63 % in 2030 and 71 % by 2050 in low and middle-income countries [4].

Depression, on the other hand, is a highly prevalent psychiatric disorder among the older population and is the most frequent cause of emotional suffering in later years of life [5, 6]. Late-life depression (LLD) is a significant public health concern as it leads to functional decline, physical disability, and increased health care usage [7, 8]. It negatively impacts physical and psychological health and quality of life [9, 10]. As per epidemiological data, about 11–35 % of older adults experience clinically significant depressive symptoms [5, 11]. However, these rates are even higher in clinical settings [12]. Depressive symptoms are more frequent among the oldest old population, but the higher frequency is explained by the factors like aging, physical disability, and cognitive impairment [5, 13]. Nevertheless, from a diagnostic perspective, LLD is often under-recognized, under-reported, and under-treated [14].

A growing body of literature suggests that depression is a prodrome to dementia and plays a major role in the development of long-term cognitive disabilities [15]. Individuals with LLD frequently present with several cognitive complaints in the clinical environment, and it is estimated that 20–50 % of older adults with LLD have cognitively impaired abilities [16, 17]. Depression and cognitive impairment are estimated to co-occur among 25 % of older individuals aged 85 and above [18]. According to Kingston et al. [19], over the next two decades, the proportion of older adults with both cognitive impairment and depressive symptoms will increase. The relationship between depression and cognitive impairment is however complicated. Depressive symptoms frequently lead to complaints about deteriorating cognitive function, and cognitive deficiencies lead to complaints about depression [20, 21]. As a result, determining whether depression causes cognitive deterioration or cognitive impairment leads to depression is often difficult [22].

Several studies have reported that people with LLD do worse in their cognitive functioning than the people who are not depressed, with working memory, executing skills, and information processing capacity being the most frequently affected [23, 24]. It has also been observed that many older adults who are cognitively affected after a depressive disorder continue to be cognitively disabled even after the illness has passed [25]. Nevertheless, depressive symptoms may result in the progression of mild cognitive impairment to dementia [26]. LLD has been linked to an elevated risk of dementia, including Parkinson’s disease and Alzheimer’s disease, according to research [27, 28]. On the other hand, LLD in many cases is also considered to have a significant negative impact on the overall functioning of older individuals, giving rise to the controversial condition of ‘pseudodementia’ [29].

In India, little is known about the prevalence of depression and cognitive impairment in older adults. However, most of the research in this field is confined to a limited geographical context and consequently, sample sizes are small to yield precise estimates. Older individuals with impaired cognitive function often have more than one risk factor. It is important to identify those risk factors and determine their association with cognitive impairment especially in the Indian context where illiteracy exists on an appalling scale. Therefore, we aimed to estimate the prevalence of LLD and cognitive impairment among older adults aged 60 and above. Furthermore, it was of interest to explore the relationship between LLD and cognitive impairment among the older population in India.

## Methods

### Data source

Data for this study was derived from the Longitudinal Ageing Study in India (LASI) Wave 1 (2017-18) [30]. The LASI is a full-scale national survey of scientific investigation of the health, economic, and social determinants and consequences of population aging in India. This nationally representative survey included over 72,000 older adults aged 45 and above and their spouses irrespective of their ages, across all states and union territories in India. The survey adopted a multistage stratified area probability cluster sampling design to arrive at the eventual units of observation: older adults aged 45 and above. It was conducted with a three-stage sampling design in rural areas and a four-stage sampling design in urban areas. In each state/UT, the first stage involved the selection of Primary Sampling Units (PSUs), that is, sub-districts (Tehsils/Talukas), and the second stage involved the selection of villages in rural areas and wards in urban areas in the selected PSUs. In rural areas, households were selected from selected villages in the third stage. However, sampling in urban areas involved an additional stage. Specifically, in the third stage, one Census Enumeration Block (CEB) was randomly selected in each urban area and in the fourth stage, households were selected from this CEB. The detailed methodology with the complete information on the survey design and data collection was published in the survey report [30]. The present study focused on the eligible respondents aged 60 years and above. The total sample size for the present study was 31,464 (15,098 male and 16,366 female) older individuals aged 60 years and above.

### Outcome variables

There were two outcome variables used in the study. Both the outcome variables were dichotomized in the analyses.

1. Cognitive impairment was measured through five broad domains (memory, orientation, arithmetic function, executive function, and object naming). The cognitive measures in the LASI were derived from The University of Michigan Health and Retirement Study (HRS). Memory was measured using immediate word recall (0–10 points) and delayed word recall (0–10 points); orientation was measured using the time (0–4 points) and place (0–4 points) measure; arithmetic function was measured through backward counting (0–2 points), serial seven (0–5 points), and computation method (0–2); executive function was measured through paper folding (0–3) and pentagon drawing method (0–1); and finally, an object naming was conducted (0–2) among the study participants. A composite score of 0–43 was computed using the domain-wise measure with a higher score representing better cognitive functioning. The lowest 10th percentile is used as a proxy measure of cognitive impairment [30]. In our study, the respondents who received assistance during the cognition module were excluded from the analysis.
2. The major depression among the older adults with symptoms of dysphoria was calculated using the CIDI-SF (Composite International Diagnostic Interview- Short Form) with a score of 3 or more out of 10 symptoms (as the lowest 10th percentile is used as a proxy measure of major depression among older adults) leading to a 0.55 Probability of CIDI caseness of major depression. However, a cut-off of 5/10 symptoms (0.89 Probability of CIDI caseness of major depression) and 7/10 symptoms (0.91 Probability of CIDI caseness of major depression) had also been chosen for the sensitivity analyses in the present study (results given in the supplementary file). The scale estimates a probable psychiatric diagnosis of major depression and has been validated in field settings especially by non-clinicians in general population surveys and widely used in population-based health surveys [31]. Cronbach’s alpha indicated that CIDI-SF has excellent reliability (α = 0.8).

The questions which were used to assess the depression were as follow:

1. During the last 12 months, was there ever a time when you felt sad, blue, or depressed for two weeks or more in a row?
2. Please think of the two weeks during the last 12 months when these feelings were worst. During that time, did the feelings of being sad, blue, or depressed usually last all day long, most of the day, about half the day, or less than half the day?
3. During those two weeks, did you feel this way every day, almost every day, or less often than that?
4. Did you lose interest in most things?
5. Did you ever feel more tired out or low in energy than is usual for you?
6. Did you lose your appetite?
7. During the same two-week period, did you have a lot more trouble concentrating than usual?
8. People sometimes feel down on themselves and no good or worthless. During those two weeks, did you feel this way?
9. Did you think a lot about death – either your own, someone else’s, or death in general – during those two weeks?
10. Did you have more trouble falling asleep than you usually do during those two weeks?

### Explanatory variables

1. Age was coded as ‘young old’ (60–69 years), ‘old-old (70–79 years), and ‘oldest-old (80 + years).
2. Sex was coded as ‘male’ and ‘female’.
3. Educational status was coded as ‘no education/primary not completed’, ‘primary’, ‘secondary’, and ‘higher’.
4. Marital status was recoded as ‘currently in marital union’ and ‘not in a marital union’ which includes ‘never married, divorced, separated & widowed.
5. Working status was coded as 'never/not working', ‘currently working’, and ‘retired’.
6. Living arrangement was recoded as ‘alone’, ‘with spouse’, and ‘others’.
7. Community involvement was measured through the question “Are you a member of any of the organizations, religious groups, clubs, or societies? And it was recoded as ‘no’ for no community involvement and ‘yes’ for representing community involvement.
8. Physical activity status was assessed through the question “How often do you take part in sports or vigorous activities, such as running or jogging, swimming, going to a health center or gym, cycling, or digging with a spade or shovel, heavy lifting, chopping, farm work, fast bicycling, cycling with loads?” the responses were recoded as ‘yes’ (every day, more than once a week, once a week, one to three times in a month), and ‘no’ (never) [30].
9. Self-rated health was coded as ‘good’ which includes very good, good, and fair; whereas, ‘poor’ includes poor and very poor [32].
10. Activities of Daily Living (ADL) is a term used to refer to normal daily self-care activities (such as movement in bed, changing position from sitting to standing, feeding, bathing, dressing, grooming, personal hygiene, etc.). The ability or inability to perform ADLs is used to measure a person’s functional status, especially in the case of people with disabilities and the ones in their older ages [33]. It is coded as 'no' and 'yes' representing having difficulty in ADL.
11. Difficulty in IADL (Instrumental Activities of Daily Living) was coded as ‘no’ and ‘yes’. Activities of daily living that are not necessarily related to the fundamental functioning of a person, but they let an individual live independently in a community. These tasks are necessary for independent functioning in the community. Respondents were asked if they were having any difficulties that were expected to last more than three months, such as preparing a hot meal, shopping for groceries, making a telephone call, taking medications, doing work around the house or garden, managing money (such as paying bills and keeping track of expenses), and getting around or finding an address in unfamiliar places [34].
12. The monthly per capita consumption expenditure (MPCE) quintile was assessed using household consumption data. Sets of 11 and 29 questions on the expenditures on food and non-food items, respectively, were used to canvas the sampled households. Food and non-food expenditures have been standardized to the 30-days reference period. The MPCE is computed and used as the summary measure of consumption. The variable was then divided into five quintiles i.e., from poorest to richest.
13. Religion was recoded as ‘Hindu’, ‘Muslim’, and ‘Others’.
14. Caste was recoded as ‘Scheduled Caste/Scheduled Tribe’ (SC/ST), ‘Other Backward Class’ (OBC), and ‘Others’.
15. Place of residence was coded as ‘rural’ and ‘urban’.
16. The regions of India were coded as ‘North’, ‘Central’, ‘East’, ‘Northeast’, ‘West’, and ‘South’ [35].

### Statistical analyses

Descriptive statistics along with cross-tabulation were presented in the present study. Additionally, binary logistic regression analyses [36] were performed to establish the association between the outcome variables and explanatory variables.

The binary logistic regression model is usually put into a more compact form as follows:

$$\text{L}\text{o}\text{g}\text{i}\text{t} \left[\text{P}\left(\text{Y}=1\right)\right]={\beta }_{0}+\beta *X$$

The parameter ${\beta }_{0}$ estimates the log odds of cognitive impairment for the reference group, while $\beta$ estimates the maximum likelihood, the differential log odds of major depression and cognitive impairment associated with a set of predictors X, as compared to the reference group.$< mathdollar>$

Model-1 provides the estimates of cognitive impairment by major depression and is adjusted for all other explanatory variables in the study. Model-2 which is adjusted for all the covariates represents the interaction effect [37–39] of major depression with the place of residence on cognitive impairment among older adults to find out the rural-urban gradient in the association of depression and cognitive impairment.

The complex survey design effects were adjusted by using STATA *svyset* and *svy* commands. The whole statistical analyses were performed by using STATA version 14 [40].

## Results

### Socio-demographic profile of the study participants

Table 1 represents the socio-demographic profile of the older adults aged 60 and above in India. The share of the young-old population was nearly 59 % while the share of the old-old and oldest-old population was around 30 and 11 % respectively. In the study sample, about 53 % of the older adults were females. Nearly 68 % of the older adults were illiterate or their primary education was incomplete, whereas, only 7 % were highly educated. Besides, around 30 % of the study participants belonged to urban areas against 70 % who were rural residents.

**Table 1:** Socio-economic profile of the study sample

**Background Factors**	**Sample**	**Percentage**
**Age (in years)**		
Young old (60–69)	18,410	58.5
Old old (70–79)	9,501	30.2
Oldest old (80+)	3,553	11.3
**Sex**		
Male	14,931	47.5
Female	16,533	52.6
**Education**		
No education/primary not completed	21,381	68
Primary	3,520	11.2
Secondary	4,371	13.9
Higher	2,191	7
**MPCE quintile**		
Poorest	6,829	21.7
Poorer	6,831	21.7
Middle	6,590	21
Richer	6,038	19.2
Richest	5,175	16.5
**Religion**		
Hindu	25,871	82.2
Muslim	3,548	11.3
Others	2,045	6.5
**Caste**		
SC/ST	8,505	27.1
OBC	14,231	45.2
Others	8,729	27.7
**Place of residence**		
Rural	22,196	70.6
Urban	9,268	29.5
**Region**		
North	3,960	12.6
Central	6,593	21
East	7,439	23.6
Northeast	935	3
West	5,401	17.2
South	7,136	22.7
**Total**	31,464	100

*MPCE* Monthly per capita consumption expenditure; *ADL* Activities of daily living; *IADL* Instrumental activities of daily living

### Bivariate and logistic regression analyses of LLD among older adults

Table 2 gives a representation of the bivariate and logistic regression estimates for LLD among older adults. The overall prevalence of LLD in this sample was 8.7 %. Nearly 11 % of elderly aged 80 and above were suffering from LLD against 8.4 % of older adults aged 60–69 of which the Chi2 test showed no significance. The prevalence rate of LLD was greater among females (9.7 %) than the males (7.5 %). The percentage of LLD was highest among older adults with less education (9.5 %). Further, more than 10 % of older adults with LLD were not in a marital union. Nearly 13.5 % of the older adults who lived alone reported suffering from LLD.

**Table 2:** Bivariate and logistic regression estimates for major depression among older adults in India

**Variables**	**%**	***p* < 0.05**	**AOR (95 % CI)**
**Age (in years)**			
Young old (60–69)	8.41		Ref.
Old old (70–79)	8.42		0.788*** (0.676–0.918)
Oldest old (80+)	10.79		0.805* (0.623–1.040)
**Sex**		*	
Male	7.5		Ref.
Female	9.71		1.136 (0.967–1.333)
**Educational status**		*	
No/primary education	9.55		Ref.
Secondary	6.39		0.903 (0.745–1.095)
Higher	5.56		0.771 (0.561–1.059)
**Marital status**		*	
Currently in union	7.77		Ref.
Not in union	10.13		1.211** (1.020–1.438)
**Living arrangement**		*	
Alone	13.51		Ref.
With spouse	8.56		0.785 (0.570–1.082)
Others	8.32		0.718** (0.548–0.939)
**Working status**		*	
Never/Not working	9.17		Ref.
Currently working	7.87		1.190** (1.007–1.406)
Retired	7.74		1.302* (0.954–1.776)
**Self-rated health**		*	
Good	6.19		Ref.
Poor	16.42		2.586*** (2.237–2.989)
**ADL difficulty**		*	
No	6.69		Ref.
Yes	15.34		1.736*** (1.451–2.077)
**IADL difficulty**		*	
No	5.58		Ref.
Yes	12.06		1.694*** (1.429–2.007)
**MPCE quintile**		*	
Poorest	8.88		Ref.
Poorer	7.92		0.916 (0.759–1.105)
Middle	8.17		1.035 (0.825–1.298)
Richer	8.74		1.111 (0.900–1.371)
Richest	9.92		1.337*** (1.088–1.642)
**Religion**		*	
Hindu	8.6		Ref.
Muslim	9.63		1.049 (0.843–1.305)
Others	7.94		0.93(0.53,1.64)
**Caste**		*	
SC/ST	8.48		Ref.
OBC	9.25		1.282*** (1.080–1.520)
Others	7.9		1.056 (0.873–1.278)
**Place of residence**		*	
Rural	9.62		Ref.
Urban	6.34		0.826** (0.698–0.978)
**Region**		*	
North	6.8		Ref.
Central	14.53		2.275*** (1.840–2.813)
East	8.28		1.052 (0.864–1.281)
Northeast	5.63		0.813 (0.600–1.101)
West	5.82		0.599*** (0.474–0.758)
South	7.69		1.078 (0.856–1.358)
**Total**	8.67		

%: Percentage; Ref: Reference category; *if *p* < 0.05, **if *p* < 0.01, ***if *p* < 0.001; *AOR* Odds Ratio Adjusted for all covariates; *ADL* Activities of daily living; *IADL* Instrumental activities of daily living; *MPCE* Monthly per capita consumption expenditure

Moreover, multi-variable analysis shows that the oldest old participants were 20 % less likely to suffer from LLD [OR: 0.80, CI: 0.62–1.04] in comparison to the young-old adults. The older adults who were not in a marital union were 21 % more likely to suffer from depression in late life [OR: 1.21, CI: 1.02–1.44] in comparison to the older adults who were currently married. Notably, the odds of depression were lower among older adults who lived with their children and others [OR: 0.72, CI: 0.55–0.94] with respect to the older adults who lived alone. The older adults who were economically active or retired were 19 and 29 % significantly more likely to suffer from LLD in comparison to the older adults who were economically inactive. Among older individuals who have rated their health status as poor were 2.59 times more likely to suffer from LLD [OR: 2.59, CI: 2.24–2.99] as compared to their counterparts. The older adults who had difficulty in ADL and IADL were 74 % [OR: 1.74, CI: 1.45–2.08] and 69 % [OR: 1.69, CI: 1.43–2.01] significantly more likely to suffer from LLD in comparison to the older adults who had no ADL and IADL difficulty respectively. Interestingly, the odds of depression were significantly higher among the older adults who belonged to the highest MPCE quintile [OR: 1.34, CI: 1.09–1.64] in reference to the older people who were from the poorest MPCE quintile. Besides, urban dwellers had lower odds of suffering from LLD [OR: 0.83, CI: 0.70–0.98] compared to their rural counterparts.

### Percentage of depressed and cognitively impaired older adults by age, sex, and place of residence

**Fig. 1:**
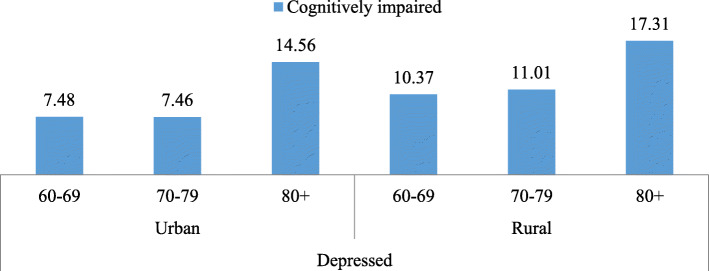
Percentage of older adults who are depressed and cognitively impaired by age and place of residence

Figure 1 depicts the percentage of cognitive impairment among older adults who were depressed by their age and place of residence. In the case of both urban and rural areas, the oldest old individuals aged 80 and above had the highest percentage of cognitive impairment compared to all other age groups.

**Fig. 2:**
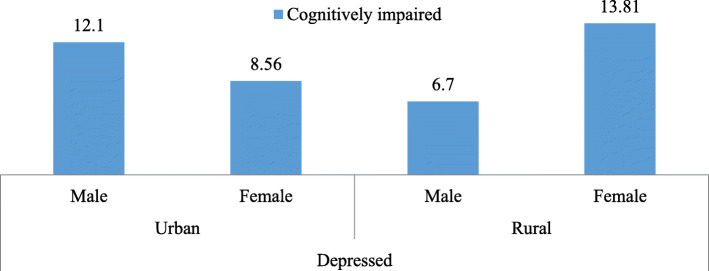
Percentage of older adults who are depressed and cognitively impaired by sex and place of residence

Figure 2 reveals the percentage of cognitive impairment among older adults who were depressed by their sex and place of residence. Among urban dwellers, 12.1 % males were cognitively impaired against 8.6 % females whereas, in the case of rural areas, females (13.8 %) had shown a greater percentage for cognitive impairment.

### Bivariate analyses and logistic regression estimates of cognitive impairment among older adults

Table 3 depicts the share of older adults who suffered from cognitive impairment. According to the analysis, the prevalence of cognitive impairment for the current sample was 13.7 %. It was found that around 18 % of the older adults who were depressed also suffered from cognitive impairment while 17 % of the older adults suffering from cognitive impairment were rural residents. About 28 % of the oldest-old individuals were suffering from cognitive impairment. Older women had a substantially higher percentage of cognitive impairment (20 %) than men (7.1 %).

**Table 3:** Bivariate and logistic regression estimates for cognitive impairment by background characteristics among older adults in India

**Variables**	**%**	*p* < 0.05	**AOR (95 % CI)**	**AOR (95 % CI)**
			**Model 1**	**Model 2**
**Depression**		*		
No	13.23		Ref.	
Yes	18.26		1.224** (1.015–1.476)	
**Place of residence**		*		
Urban	6.69		Ref.	
Rural	16.67		2.120*** (1.732–2.595)	
**Age (in years)**		*		
Young old (60–69)	10.03		Ref.	Ref.
Old old (70–79)	16.77		1.635*** (1.395–1.916)	1.634*** (1.395–1.915)
Oldest old (80+)	27.76		2.922*** (2.396–3.564)	2.921*** (2.396–3.562)
**Sex**		*		
Male	7.14		Ref.	Ref.
Female	20.03		2.264*** (1.947–2.634)	2.266*** (1.948–2.636)
**Marital status**		*		
Currently in union	9.68		Ref.	Ref.
Not in union	20.77		1.536*** (1.307–1.806)	1.536*** (1.306–1.805)
**Living arrangement**		*		
Alone	19.07		Ref.	Ref.
With spouse	11.92		1.605*** (1.191–2.163)	1.604*** (1.190–2.162)
Others	13.73		1.174 (0.927–1.487)	1.174 (0.927–1.487)
**Working status**		*		
Never/Not working	17.08		Ref.	Ref.
Currently working	9.98		0.773*** (0.662–0.903)	0.773*** (0.662–0.903)
Retired	2.24		0.526*** (0.333–0.833)	0.527*** (0.333–0.833)
**Community involvement**				
No	14.12	*	Ref.	Ref.
Yes	5.24		0.604*** (0.433–0.842)	0.603*** (0.432–0.842)
**Physical activity**				
No	14.87	*	Ref.	Ref.
Yes	6.85		0.741* (0.536–1.025)	0.740* (0.535–1.025)
**Educational status**		*		
No/primary education	18.79		Ref.	Ref.
Secondary	1.05		0.0879*** (0.0600–0.129)	0.0881*** (0.0601–0.129)
Higher	0.42		0.0616*** (0.0254–0.149)	0.0616*** (0.0254–0.149)
**MPCE quintile**		*		
Poorest	18.55		Ref.	Ref.
Poorer	15.75		0.905 (0.757–1.082)	0.906 (0.758–1.082)
Middle	12.46		0.716*** (0.600–0.853)	0.716*** (0.601–0.854)
Richer	10.88		0.645*** (0.530–0.785)	0.645*** (0.530–0.786)
Richest	9.81		0.678*** (0.513–0.896)	0.679*** (0.514–0.897)
**Religion**		*		
Hindu	13.37		Ref.	Ref.
Muslim	14.62		1.261* (1.007–1.579)	0.892 (0.714–1.113)
Others	15.72		1.121 (0.898–1.399)	1.127 (0.837–1.515)
**Caste**		*		
SC/ST	19.74		Ref.	Ref.
OBC	12.03		0.643*** (0.554–0.747)	1.464*** (1.230–1.742)
Others	10.8		0.683*** (0.574–0.813)	0.941 (0.789–1.123)
**Region**		*		
North	13.02		Ref.	Ref.
Central	14.27		1.007 (0.823–1.234)	1.009 (0.824–1.235)
East	14.78		1.099 (0.906–1.333)	1.100 (0.907–1.334)
Northeast	15.35		1.317** (1.061–1.635)	1.317** (1.061–1.635)
West	10.97		0.974 (0.781–1.213)	0.975 (0.782–1.214)
South	15.02		1.541*** (1.256–1.892)	1.542*** (1.257–1.893)
**Depression # Place of residence**				
No # Urban				Ref.
Yes # Urban				1.390 (0.818–2.362)
No # Rural				2.148*** (1.729–2.668)
Yes # Rural				2.579*** (1.951–3.409)
**Total**	13.66			

%: Percentage; Ref: Reference category; *if *p* < 0.05, **if *p* < 0.01, ***if *p* < 0.001; *AOR* Odds Ratio Adjusted for all covariates; *MPCE* Monthly per capita consumption expenditure

In the logistic regression analysis, it was found that the older adults who were depressed were 22 % significantly more likely to suffer from cognitive impairment [OR: 1.22, CI: 1.02–1.48] in reference to the older adults who were not depressed. Also, the older population of rural areas was significantly more likely to be cognitively impaired [OR: 2.12, CI: 1.73–2.06] than their urban counterparts. In comparison to the population in the age group of 60–69, elderly aged 80 and above were nearly three times more likely to suffer from cognitive impairments [OR: 2.92, CI: 2.40–3.56]. Importantly, females were 2.3 times more likely to develop cognitive impairment [OR: 2.26, CI: 1.95–2.63] as compared to men. The odds of cognitive impairment were significantly higher among the older adults who were living with their spouse in comparison to the older adults who were living alone [OR: 1.61, CI: 1.19–2.16]. The older adults who were economically active or retired were 23 % [OR: 0.77, CI: 0.66–0.90] and 47 % [OR: 0.53, CI: 0.33–0.83] respectively less likely to develop cognitive impairment in comparison to the older adults who were economically inactive. Further, the odds of cognitive impairment were lower among older adults who were physically active [OR: 0.74, CI: 0.54–1.02] and had community involvement [OR: 0.60, CI: 0.43–0.84] as compared to their reference categories. According to the results, the odds of cognitive impairment decreased with the increase in educational level and MPCE quintile.

Model-2 represents the interaction effect of major depression along with the place of residence of older individuals on cognitive impairment. Older adults who were depressed and belonged to rural areas were 2.58 times [AOR: 2.58, CI: 1.95–3.41] significantly more likely to be cognitively impaired than those who were not depressed and resided in urban areas.

## Discussion

The current analysis of the cross-sectional study shows that the prevalence estimate of LLD in a representative sample of older adults aged 60 and above in India is 8.7 %. The bivariate analysis shows no significance in the prevalence of LLD with an increasing age. This is in variance with the earlier findings that increasing age is associated with more neurological or chronic degenerative disorders [41]. However, on contrary, the multivariate results show a negative association of LLD with increasing age. In accordance with earlier studies, the results indicate that older adults who are currently not in union and living alone are more likely to suffer from depression [42–44]. Also, as evidence suggests, solo living and widowhood may create a situation of loneliness, isolation, and loss of roles that can lead to depression [45–47].

With regard to work status, retirement is significantly associated with LLD. This is in line with the studies from Ethiopia, Sudan, and Korea [48–50]. This could be explained as the retired person may not have adequate opportunity to interact with other people to share their ideas and feelings. This brings a sense of loss, leaving a person feeling isolated and struggling to understand what their value is and such feelings might contribute to the development of depressive symptoms in their older ages [51]. In concordance with previous studies [52–54], the current study also showed that older individuals with poor self-rated health and low functional ability in ADL and IADL are at higher risk of suffering from LLD. It is also evident from the study that individuals living in rural areas are more likely to suffer from depression in later years of life. This result is consistent with the findings from a study conducted in China showing a higher proportion of depressive symptomatology and feelings of loneliness among rural resident older adults [55].

Studies in various developed and developing countries have proven that there is a higher prevalence of cognitive impairment in low-and middle-income countries [56–58]. Further, various evidence in India has also shown an upward trend in the prevalence of cognitive impairment since the 1990s [59, 60]. In this study, the overall prevalence of cognitive impairment among the older population is found to be substantially high with 13.7 %. Studies conducted in different parts of the country also have documented nearly similar prevalence [59, 61].

Several studies have shown that the risk of cognitive impairment increases with age [2, 62]. In this study, cognitive impairment is significantly higher among older adults aged 70 and above compared to those in the age group of 60–69 years and highest in the 80 plus population. Aging is associated with various changes in brain structure and function [63, 64]. More so, the brain volume starts shrinking after the age of 40, and the rate increases after the age of 70 which leads to a decline in executive functioning resulting in cognitive impairment [65]. The analysis revealed that females are significantly more likely to develop cognitive impairment than males, which corresponds to the study by Khanna & Metgud in 2020 [66]. A possible explanation for the high prevalence of cognitive impairment among older women could be the loss of estrogen hormone after menopause. Some researchers have reported that declines in estrogen level could lead to deficits in the cognitive ability of post-menopausal women [67, 68]. Further, according to this study, the risk of cognitive impairment is substantially higher among older adults living in rural areas as compared to their counterparts in urban areas and is similar to the findings in earlier studies [38, 69, 70].

Older adults who are currently in a marital union or living with their spouses are found to be at greater risk of cognitive impairment. However, this has not been reported in other studies [38, 62] and needs further investigation. Besides, education and socioeconomic status have a strong negative association with cognitive impairment and the results are parallel to a study conducted in India [71], supporting the brain reserve hypothesis that more years of education translates into greater cognitive reserve [70, 72, 73]. Further, high socio-economic status means greater access to healthcare services, high nutritious food intake as well as higher levels of social activities, which have been proven to improve cognitive functioning [74, 75]. A study conducted in Beijing has documented that less social interaction is an independent risk factor for cognitive impairment [76]. Similarly, according to our study, the prevalence of cognitive impairment was higher among older individuals with no community involvement.

Moreover, the study’s results back up the theory that LLD can be a risk factor for the development of cognitive disability in older people. According to a hypothesis, late-life depressive symptoms could indicate an underlying neuropathologic disorder that contributes to cognitive deterioration over time [77, 78]. Depressive symptoms have also been linked to cognitive impairment, according to many studies [6, 42, 79]. Mild depression, on the other hand, does not result in significant cognitive dysfunction, whereas persistent depression in its most extreme form does [80]. Several pieces of literature have suggested that LLD sufferers have more cognitive deficits than those suffering from early-onset depression [24, 81]. Depression-related executive-type cognitive impairments can explain why depressed persons are more disabled [82]. Although geriatric depression may increase the risk of cognitive impairments, studies have also demonstrated co-occurrence of depression, cognitive functioning, and dementia [78, 83], and possible reverse causation between them [84, 85]. Similarly, the prevalence of depression in dementia has been reported to range between 9 and 68 % [86], indicating the need for further investigation of the observed pathways. Further, in our study, an important association between depression and rural residence on cognitive impairment is observed in the regression model with interaction analysis. The older adults suffering from LLD and living in rural areas exhibit greater cognitive deficits than those living in urban areas. The result is in line with various studies that suggested that elderly living in urban areas have lower dementia rates and do better on cognitive tests [82, 87]. Though the association is not well understood, some theories explain the disparity by pointing to rural dwellers’ lower educational quality and reduced access to public and health services [87].

The current study has major limitations to be acknowledged. Importantly, the study was cross-sectional; therefore, any causal pathways cannot be definitively determined from the results, rather reverse causation stands as a possibility in many of the cases including depression and cognitive impairment. The forthcoming followed-up wave 2 of the LASI data will provide greater scope to understand the causal relationship between depressive symptoms and cognitive impairments among older Indian adults. Also, the findings can be somewhat biased towards a cognitively well-functioning population due to a substantially higher illiteracy rate and the unavailability of information on institutionalized older people and exclusion of proxy-assisted respondents, and a small portion of cognitive data that was missing in the analyses. Hence, it suggests that the present results may not generalize to individuals with severely impaired cognitive status, as they were not adequately sampled in this study. However, this study has several strengths too. First, the data were derived from a large, national probability sample of older people aged 60 and above, enhancing external validity and generalizability of the current findings. Again, the measure of cognition was comprehensive and multi-faceted, and it tapped into participants’ memory capabilities as well as their mental status.

## Conclusions

Because of the strong association between the two, cognitive impairment and LLD have become major health concerns among older adults. Since life expectancy is growing, older age is one of the most important non-modifiable risk factors for cognitive impairment and should be included in health interventions. Furthermore, depression is linked to an increased risk of cognitive decline and dementia; therefore, failing to diagnose and treat LLD in later life may have significant health implications. Moreover, treatment under the care of a cognitive neurologist or geriatric psychiatrist is recommended for people with LLD and cognitive disability due to both the disorders' complex existence.

## Supplementary Information

**Additional file 1.**

## Abbreviations

LLD: Late-life depression.
AOR: Adjusted Odds Ratio.
CI: Confidence Interval.
MPCE: Monthly per capita consumption expenditure.
ADL: Activities of daily living.
IADL: Instrumental activities of daily living.
