# Supplementary material for: Association of late-life depression with cognitive impairment: evidence from a cross-sectional study among older adults in India
Source: BMC Geriatr. 2021 Jun 15;21:364. doi: 10.1186/s12877-021-02314-7 (PMC8204463; doi:10.1186/s12877-021-02314-7)
Supplement: Supplementary file 1 — Additional file 1. [file 12877_2021_2314_MOESM1_ESM.docx]

**Supplementary file**

| Odds ratio of cognitive impairment observed in the sensitivity analyses with different cut-off points for depressive symptoms (5/10 and 7/10) | | | |
| --- | --- | --- | --- |
| **Variables** |  | **AOR (95% CI)^1^** | **AOR (95% CI)^2^** |
| **Depression** |  |  |  |
| No | |  |  |
| Yes | | 1.230** (1.018 - 1.487) | 1.237** (1.014 - 1.508) |
| **Place of residence** |  |  |  |
| Urban | |  |  |
| Rural | | 2.177*** (1.763 - 2.690) | 2.174*** (1.760 - 2.685) |
| **Age (in years)** |  |  |  |
| Young old (60-69) | |  |  |
| Old-old (70-79) | | 1.625*** (1.387 - 1.905) | 1.625*** (1.386 - 1.904) |
| Oldest-old (80+) | | 2.867*** (2.355 - 3.488) | 2.868*** (2.357 - 3.491) |
| **Sex** |  |  |  |
| Male | |  |  |
| Female | | 2.261*** (1.943 - 2.630) | 2.262*** (1.945 - 2.632) |
| **Marital status** |  |  |  |
| Currently in union | |  |  |
| Not in union | | 1.527*** (1.298 - 1.797) | 1.529*** (1.299 - 1.798) |
| **Living arrangement** |  |  |  |
| Alone | |  |  |
| With spouse | | 1.628*** (1.209 - 2.192) | 1.625*** (1.207 - 2.188) |
| Others | | 1.183 (0.935 - 1.496) | 1.181 (0.934 - 1.495) |
| **Working status (last one year)** | |  |  |
| Never/Not | |  |  |
| Yes | | 0.859 (0.715 - 1.033) | 0.861 (0.716 - 1.035) |
| Retired | | 0.526*** (0.331 - 0.835) | 0.525*** (0.331 - 0.834) |
| **Community involvement** |  |  |  |
| No | |  |  |
| Yes | | 0.605*** (0.433 - 0.843) | 0.604*** (0.433 - 0.843) |
| **Physical activity** |  |  |  |
| No | |  |  |
| Yes | | 0.799** (0.647 - 0.988) | 0.798** (0.646 - 0.986) |
| **Educational status** | |  |  |
| No/primary education | |  |  |
| Secondary | | 0.0870*** (0.0595 - 0.127) | 0.0870*** (0.0595 - 0.127) |
| Higher | | 0.0591*** (0.0245 - 0.143) | 0.0591*** (0.0245 - 0.143) |
| **MPCE** | |  |  |
| Poorest | |  |  |
| Poorer | | 0.904 (0.757 - 1.080) | 0.903 (0.756 - 1.080) |
| Middle | | 0.717*** (0.601 - 0.855) | 0.717*** (0.601 - 0.855) |
| Richer | | 0.640*** (0.526 - 0.780) | 0.640*** (0.525 - 0.780) |
| Richest | | 0.678*** (0.513 - 0.896) | 0.677*** (0.512 - 0.895) |
| **Religion** |  |  |  |
| Hindu | |  |  |
| Muslim | | 1.268** (1.011 - 1.590) | 1.268** (1.011 - 1.590) |
| Others | | 1.120 (0.897 - 1.399) | 1.121 (0.897 - 1.400) |
| **Caste** |  |  |  |
| SC/ST | |  |  |
| OBC | | 0.641*** (0.552 - 0.745) | 0.641*** (0.551 - 0.745) |
| Others | | 0.677*** (0.569 - 0.806) | 0.677*** (0.569 - 0.806) |
| **Region** | |  |  |
| North | |  |  |
| Central | | 1.001 (0.817 - 1.226) | 1.001 (0.817 - 1.227) |
| East | | 1.106 (0.913 - 1.339) | 1.106 (0.913 - 1.339) |
| Northeast | | 1.351*** (1.092 - 1.672) | 1.354*** (1.094 - 1.676) |
| West | | 0.982 (0.791 - 1.220) | 0.985 (0.793 - 1.223) |
| South | | 1.575*** (1.284 - 1.931) | 1.576*** (1.285 - 1.932) |
| ***^1^*** *Cut off of 5/10 depressive symptoms with a 0.89 Probability of CIDI Caseness of Major depression;* ***^2^*** *Cut off of 7/10 depressive symptoms with a 0.91 Probability of CIDI Caseness of Major depression; *if p<0.05, **if p<0.01, ***if p<0.001; AOR: Odds Ratio Adjusted for all covariates; MPCE: Monthly per capita expenditure* | | | |
